# Management of sequelae of Kawasaki disease in adults

**DOI:** 10.21542/gcsp.2017.31

**Published:** 2017-10-31

**Authors:** John B. Gordon, Jane C. Burns

**Affiliations:** 1 San Diego Cardiac Center and Sharp Memorial Hospital, San Diego, CA, USA; 2 Dept of Pediatrics, University of California San Diego, and Rady Children’s Hospital, San Diego, CA, USA

## Abstract

**Background:** A growing population of young adults is presenting to cardiologists with late manifestations of Kawasaki disease (KD) that include cardiomyopathy, ischemia, and infarction. The management of these conditions differs in important ways from atherosclerotic heart disease, and yet there is little awareness in the adult cardiology community regarding the special challenges posed by the cardiovascular sequelae of KD.

**Methods:** Observations were made on a population of 140 adult KD patients enrolled in the San Diego Adult KD Collaborative Study.

**Results:** Coronary artery aneurysms resulting from KD in childhood are associated with a high risk of thrombosis and stenosis at the inlet or outlet of the aneurysm. These aneurysms are often highly calcified and may contain a large thrombus burden that may obscure the true size of the aneurysm. Pitfalls in the management of these patients stem largely from failure to recognize the nature of the lesions, which leads to attempts to dilate highly calcified stenotic segments and undersizing of stents. Intravascular ultrasound is helpful in appreciating the true dimensions of the aneurysm, which may be filled with thrombus. Thrombolysis and use of anti-platelet agents followed by systemic anti-coagulation are appropriate management strategies for patients presenting with acute infarction. Bypass grafting with the internal thoracic arteries can be a successful strategy, but care must be taken to avoid competitive flow through the native vessel leading to graft failure. In contrast to the individuals who developed coronary artery aneurysms, young adults who had documented normal echocardiograms associated with their acute KD in childhood and who have no evidence of calcium deposition in the arterial wall as assessed by computed tomography (CT) calcium score appear to have no increased cardiovascular risk in the medium term. Long-term outcomes for adults post-KD in childhood are still being defined.

**Conclusions:** KD poses special management challenges for the adult cardiologist who must recognize the unique features of the cardiovascular lesions in this growing population of patients.

## Introduction

A guiding principle in the care of adults with important coronary artery damage as a result of Kawasaki disease (KD) in childhood is *primum non nocere*. All such patients should be in follow up care with an adult cardiologist knowledgeable about the unique challenges in managing this patient population, which will be reviewed here.

## Understanding KD lesions

First, it is important to realize that once the normal arterial wall architecture has been damaged during the acute, inflammatory phase of KD, the affected segment will always have abnormal characteristics. The pathologic changes in these lesions can include both proliferation of myofibroblasts and layering of thrombus along the wall of the aneurysm. These processes, alone or in concert, contribute to progressive stenosis of the vessel lumen^1^. Based on cardiac catheterization studies, the endothelium is dysfunctional and demonstrates paradoxical vasoconstriction in response to intracoronary acetylcholine injection^2^. Intravascular ultrasound studies have documented the abnormal composition of the arterial wall, which cannot dilate under conditions of increased myocardial oxygen demand^3^. Thus even though the lumen appears normal by angiography, the fibrotic, calcified wall acts as a functional stenosis^4^. Autopsy studies have revealed a paucity of atherosclerotic changes in these damaged vessels^5^. Thus claims of accelerated atherosclerosis in these lesions based on virtual histology by IVUS are unfounded and the necrotic and calcified lesions detected by this method represent a unique injury pattern in KD that is not atherosclerosis.

The natural history of aneurysms over time is now emerging with the longest follow up period reported from Japan. These longitudinal studies have established that the highest complication rate is among patients with large aneurysms affecting both the left and right coronary arteries^6–8^. For the cohort with bilateral large aneurysms, the mid-term (10–30 yr. follow-up) cardiac event-free rate was only 36% with a cardiac event defined as death, myocardial infarction, or intervention. Thus, this is a group that requires frequent monitoring and therapies to prevent thrombosis.

Another important aspect of KD is the propensity to develop aneurysms in systemic arterial beds, most commonly the axillary and the iliac and common femoral arteries^9,10^. These lesions are generally symmetric and the largest aneurysms follow the same pattern as the coronary artery aneurysms with a high propensity to thrombose and calcify. Patients may present with ischemic symptoms including claudication or weakness in an extremity.

## Managing KD lesions

### Medical management

The consequences of arterial wall damage can manifest in various ways. One final common pathway is complete thrombosis of the aneurysm with development of collaterals. It is not uncommon to see individuals who have thrombosed one or more epicardial arteries with no associated symptoms of ischemia or myocardial infarction. This scenario is most often seen when the acute phase of KD occurred at a very young age, which seems to favor the development of collaterals^11^. These patients have essentially performed an “auto-bypass” with the growth of collaterals to supply territories at risk. Their potential for ischemia should be evaluated by stress echocardiography repeated at intervals to ensure timely recognition of changes in myocardial perfusion. However, many of these patients will do well and are not candidates for interventions or bypass surgery. Medical management with low dose aspirin and a statin to maintain vascular health are reasonable therapies in this patient population. These patients are not candidates for systemic anticoagulation unless there are aneurysms that have not thrombosed.

Computer simulations of cardiovascular hemodynamics suggest that the long ectatic segments that are commonly seen in the right coronary artery are at greatest risk for thrombosis^12^. The conditions of low wall sheer stress, high particle resonance time, and increased oscillatory index create a potent substrate for thrombosis. Computer simulations suggest that absolute diameter of the aneurysm is not the best predictor of thrombotic risk.

Data from observational studies and retrospective case series strongly support systemic anticoagulation for all children with a Z score (internal dimension of the right or left anterior descending arteries normalized for body surface area) ≥10 and all adults with an aneurysm diameter of 8 mm or greater^13,14^. However, the computer simulations suggest that long lesions of lesser diameter also pose a risk for thrombosis. Better methods are needed to assess the hemodynamic impact of these aneurysms to improve the selection of patients who should be treated with systemic anti-coagulation. Although warfarin has been widely used in the past, the new generation of oral anti-coagulants (direct thrombin inhibitors and Factor Xa inhibitors) will likely replace warfarin and enoxaparin in the near future. For now, systemic anti-coagulation with warfarin or enoxaparin coupled with anti-platelet therapy with aspirin forms the mainstay of therapy for patients with large aneurysms. The use of statins to promote endothelial cell health is also reasonable.

### Catheter-based interventions

Young adults may present acutely with unstable angina or ST-segment elevated myocardial infarction. In the absence of a known history of KD in childhood, the diagnosis of missed KD is made based upon the location and nature of the aneurysms. In two series examining angiograms of patients under 40 years of age presenting with acute coronary syndrome, 5 and 7% of patients from the U.S. and Egypt, respectively, were adjudicated as having lesions consistent with missed KD in childhood^15,16^. The characteristic proximal aneurysms with extensive calcification should be recognized as being due to antecedent KD and IVUS should be used during the attempted revascularization to correctly size the stent. The use of IIb/IIIa inhibitors and heparin are important adjuncts to establishing and maintaining patency in these arteries. All patients in this category will require long-term systemic anti-coagulation and anti-platelet therapy.

For young adults with a known history of aneurysms following acute KD, monitoring of lesions should be guided by principles established for atherosclerotic disease. Patients should be followed non-invasively with a combination of CT angiography and stress echocardiograms. Invasive catheterization should be reserved for those patients in whom an intervention is contemplated. Use of fractional flow reserve to assess the degree of stenosis and IVUS to correctly size the vessel for stenting are critical in the management of these lesions^17,18^.

When stenosis occurs, these lesions tend to be highly calcified and thus require different management strategies from typical atherosclerotic lesions. Rotational atherectomy is required to dilate these calcified segments as use of high balloon pressures with angioplasty can lead to neo-aneurysm formation^19–21^. There is limited experience with drug-eluting stents and bioresorbable scaffolds in KD, but in principle, both of these approaches should be appropriate for this patient population. A case report literature describes the use of covered stents to perform a “through-pass” procedure in which the large aneurysm is excluded by passage of a covered stent^22^.

### Surgical interventions

The use of arterial bypass grafting in adults post-KD has met with mixed success. The main pitfall is at the level of patient selection. In the absence of provocable ischemia, conservative medical management is often the best course. Adult cardiologists not familiar with the giant aneurysms that can result from KD often feel obligated to pursue aggressive strategies including surgery. Sometimes this is motivated by concern that these aneurysms will rupture. Fortunately, this is an extremely rare occurrence in adults as these lesions tend to be heavily calcified^23^. A potential complication of bypass surgery is persistent competitive flow through the native vessel, which may lead to graft failure^18^. Although most of the aneurysms in KD are located in the proximal coronary artery, aneurysms may also occur in the distal right coronary artery just proximal to the take-off of the posterior descending artery. These lesions are too distal to be bypassed using the internal thoracic artery.

The use of saphenous vein grafts in this young adult population is problematic due to the reduced patency rate of these grafts^24^. A more difficult approach using the gastroepiploic artery may be entertained. In general, the indications for bypass grafting in KD follow the same guidelines as for atherosclerotic disease including severe three-vessel disease. The largest experience with bypass grafting for KD is in pediatric patients and comes from Osaka^24^. In the hands of a highly skilled surgeon, the 20-year graft patency rate for children was 87% (95% CI, 78 to 93) for internal thoracic artery grafts (*n* = 154) and 44% (95% CI, 26 to 61) for saphenous vein grafts (*n* = 30) (*P* < 0.001). The challenging technical aspects of bypass surgery in children are not an issue for surgeons dealing with adult KD patients. However, the competitive flow issue must be carefully assessed before opting for surgical intervention.

## Future directions

Although this review has focused on the coronary artery pathology and clinical consequences of aneurysm formation, there is also emerging concern for progressive myocardial fibrosis that has been noted at autopsy and at cardiac transplantation^5^. Whether a subset of these young adults have smoldering inflammation in the arterial wall and in the myocardium that is clinically silent remains an open question. Future research should focus on imaging and biomarker studies that can elucidate on-going inflammation that could potentially benefit from targeted therapy. The role of myocardial fibrosis and arrhythmia in the sudden deaths being reported in this patient population requires further study^25^.

Clearly there is a need for an improved evidence base to guide the care and management of adults with coronary artery damage following KD in childhood. Establishment of a registry would be one way to track interventions and outcomes in this patient population. Unfortunately, no such registry has been established to date in any country. Barring KD-specific guidance, borrowing from best practice for adult atherosclerotic disease will have to suffice for the time being. It must be remembered, however, that the extensive calcification of these lesions, their proximal location, and the development of robust collateral circulation are important differences from atherosclerotic disease in this young and otherwise healthy population.

## Conflict of interest

None to declare

## Funding sources

This work supported in part by a grant from the Gordon and Marilyn Macklin Foundation to JCB.

## Author contributions

JBG evaluated all of the patients from whom the observations reported here were derived. JBG and JCB jointly drafted and revised the manuscript.

## References

[1] Orenstein JM, Shulman ST, Fox LM, Baker SC, Takahashi M, Bhatti TR, Russo PA, Mierau GW, de Chadarevian JP, Perlman EJ, Trevenen C, Rotta AT, Kalelkar MB, Rowley AH (2012). Three linked vasculopathic processes characterize Kawasaki disease: A light and transmission electron microscopic study. PLOS ONE.

[2] Yamakawa R, Ishii M, Sugimura T, Akagi T, Eto G, Iemura M, Tsutsumi T, Kato H (1998). Coronary endothelial dysfunction after Kawasaki disease: Evaluation by intracoronary injection of acetylcholine. J Am Coll Cardiol.

[3] Sugimura T, Kato H, Inoue O, Fukuda T, Sato N, Ishii M, Takagi J, Akagi T, Maeno Y, Kawano T (1994). Intravascular ultrasound of coronary arteries in children. Assessment of the wall morphology and the lumen after Kawasaki disease. Circulation.

[4] Suzuki A, Yamagishi M, Kimura K, Sugiyama H, Arakaki Y, Kamiya T, Miyatake K (1996). Functional behavior and morphology of the coronary artery wall in patients with Kawasaki disease assessed by intravascular ultrasound. J Am Coll Cardiol.

[5] Shimizu C, Sood A, Lau HD, Oharaseki T, Takahashi K, Krous HF, Campman S, Burns JC (2015). Cardiovascular pathology in 2 young adults with sudden, unexpected death due to coronary aneurysms from Kawasaki disease in childhood. Cardiovasc Pathol.

[6] Tsuda E, Abe T, Tamaki W (2011). Acute coronary syndrome in adult patients with coronary artery lesions caused by Kawasaki disease: Review of case reports. Cardiology in the Young.

[7] Tsuda E, Hirata T, Matsuo O, Abe T, Sugiyama H, Yamada O (2011). The 30-year outcome for patients after myocardial infarction due to coronary artery lesions caused by Kawasaki disease. Pediatric Cardiology.

[8] Tsuda E, Hamaoka K, Suzuki H, Sakazaki H, Murakami Y, Nakagawa M, Takasugi H, Yoshibayashi M (2014). A survey of the 3-decade outcome for patients with giant aneurysms caused by Kawasaki disease. Am Heart J.

[9] Hoshino S, Tsuda E, Yamada O (2015). Characteristics and fate of systemic artery aneurysm after Kawasaki disease. J Pediatr.

[10] Lee A, Shimizu C, Oharaseki T, Takahashi K, Daniels LB, Kahn A, Adamson R, Dembitsky W, Gordon JB, Burns JC (2015). Role of TGF-beta signaling in remodeling of non-coronary artery aneurysms in Kawasaki disease. Pediatr Dev Pathol.

[11] Onouchi Z, Hamaoka K, Kamiya Y, Hayashi S, Ohmochi Y, Sakata K, Shiraishi I, Hayano T, Fukumochi H (1993). Transformation of coronary artery aneurysm to obstructive lesion and the role of collateral vessels in myocardial perfusion in patients with Kawasaki disease. J Am Coll Cardiol.

[12] Sengupta D, Kahn AM, Kung E, Esmaily Moghadam M, Shirinsky O, Lyskina GA, Burns JC, Marsden AL (2014). Thrombotic risk stratification using computational modeling in patients with coronary artery aneurysms following Kawasaki disease. Biomechanics and Modeling in Mechanobiology.

[13] Suda K, Kudo Y, Higaki T, Nomura Y, Miura M, Matsumura M, Ayusawa M, Ogawa S, Matsuishi T (2009). Multicenter and retrospective case study of warfarin and aspirin combination therapy in patients with giant coronary aneurysms caused by Kawasaki disease. Circ J.

[14] Su D, Wang K, Qin S, Pang Y (2014). Safety and efficacy of warfarin plus aspirin combination therapy for giant coronary artery aneurysm secondary to Kawasaki disease: A meta-analysis. Cardiology.

[15] Daniels LB, Tjajadi MS, Walford HH, Jimenez-Fernandez S, Trofimenko V, Fick Jr DB, Phan HA, Linz PE, Nayak K, Kahn AM, Burns JC, Gordon JB (2012). Prevalence of Kawasaki disease in young adults with suspected myocardial ischemia. Circulation.

[16] Rizk SR, El Said G, Daniels LB, Burns JC, El Said H, Sorour KA, Gharib S, Gordon JB (2015). Acute myocardial ischemia in adults secondary to missed Kawasaki disease in childhood. Am J Cardiol.

[17] Noto N, Karasawa K, Kanamaru H, Ayusawa M, Sumitomo N, Okada T, Harada K (2002). Non-invasive measurement of coronary flow reserve in children with Kawasaki disease. Heart.

[18] Gordon JB, Daniels LB, Kahn AM, Jimenez-Fernandez S, Vejar M, Numano F, Burns JC (2016). The spectrum of cardiovascular lesions requiring intervention in adults after Kawasaki disease. JACC Cardiovasc Interv.

[19] Ino T, Akimoto K, Ohkubo M, Nishimoto K, Yabuta K, Takaya J, Yamaguchi H (1996). Application of percutaneous transluminal coronary angioplasty to coronary arterial stenosis in Kawasaki disease. Circulation.

[20] Ishii M, Ueno T, Akagi T, Baba K, Harada K, Hamaoka K, Kato H, Tsuda E, Uemura S, Saji T, Ogawa S, Echigo S, Yamaguchi T (2001). Guidelines for catheter intervention in coronary artery lesion in Kawasaki disease. Pediatr Int.

[21] Tsuda E, Miyazaki S, Yamada O, Takamuro M, Takekawa T, Echigo S (2006). Percutaneous transluminal coronary rotational atherectomy for localized stenosis caused by Kawasaki disease. Pediatr Cardiol.

[22] Di Mario C, Kilic ID, Yeh JS, Pighi M, Serdoz R, Gatzoulis MA, Dimopoulos K (2015). Exclusion of a giant aneurysm post-Kawasaki disease with novel polyurethane covered stents. Int J Cardiol.

[23] Miyamoto T, Ikeda K, Ishii Y, Kobayashi T (2014). Rupture of a coronary artery aneurysm in Kawasaki disease: A rare case and review of the literature for the past 15 years. J Thorac Cardiovasc Surg.

[24] Kitamura S, Tsuda E, Kobayashi J, Nakajima H, Yoshikawa Y, Yagihara T, Kada A (2009). Twenty-five-year outcome of pediatric coronary artery bypass surgery for Kawasaki disease. Circulation.

[25] Watanabe H, Kato M, Ayusawa M (2016). Potentially fatal arrhythmias in two cases of adult Kawasaki disease. Cardiol Young.

